# RETRACTED: Prażnowski et al. Assessment of the Road Surface Condition with Longitudinal Acceleration Signal of the Car Body. *Sensors* 2020, *20*, 5987

**DOI:** 10.3390/s26185952

**Published:** 2026-09-20

**Authors:** Krzysztof Prażnowski, Jarosław Mamala, Michał Śmieja, Mariusz Kupina

**Affiliations:** 1 Department of Vehicle, Faculty of Mechanical Engineering, Opole University of Technology, 45-271 Opole, Poland; j.mamala@po.edu.pl; 2 Faculty of Technical Sciences, University of Warmia and Mazury in Olsztyn, 10-710 Olsztyn, Poland; smieja@uwm.edu.pl; 3 Department of Mechanics and Structural Engineering, Faculty of Civil Engineering and Architecture, Opole University of Technology, 45-061 Opole, Poland; m.kupina@po.edu.pl

The journal retracts the article titled “Assessment of the Road Surface Condition with Longitudinal Acceleration Signal of the Car Body” [1], cited above.

Following publication, concerns were brought to the attention of the Editorial Office regarding the accuracy of the experiments described and the integrity of the figures presented in this study [1].

In accordance with the journal’s standard procedures, an investigation was conducted by the Editorial Office and the Editorial Board, which identified concerns regarding the reliability of the analysis, as well as image duplication between this article [1] and previously published material [2], produced by a similar authorship group, presenting different experimental conditions. Although the authors cooperated with the investigation, the explanations and supporting materials provided were not deemed sufficient by the Editorial Board to resolve the identified concerns.

As a consequence, the Editorial Board has lost confidence in the reliability of the reported findings and has decided to retract this publication in accordance with MDPI’s retraction policy.

This retraction was approved by the Editor-in-Chief of the journal *Sensors*.

The authors have been informed of this retraction but did not provide a comment on this decision.
